# A Web- and Mobile-Based Intervention for Comorbid, Recurrent Depression in Patients With Chronic Back Pain on Sick Leave (Get.Back): Pilot Randomized Controlled Trial on Feasibility, User Satisfaction, and Effectiveness

**DOI:** 10.2196/16398

**Published:** 2020-04-15

**Authors:** Sandra Schlicker, Harald Baumeister, Claudia Buntrock, Lasse Sander, Sarah Paganini, Jiaxi Lin, Matthias Berking, Dirk Lehr, David Daniel Ebert

**Affiliations:** 1 Department of Clinical Psychology and Psychotherapy Friedrich-Alexander-University Erlangen-Nürnberg Erlangen Germany; 2 Department of Clinical Psychology and Psychotherapy Philipps-University Marburg Marburg Germany; 3 Department of Clinical Psychology and Psychotherapy Ulm University Ulm Germany; 4 Department of Rehabilitationpsychology and Psychotherapy Albert-Ludwigs-University Freiburg Freiburg Germany; 5 Department of Sport and Sport Science Albert-Ludwigs-University Freiburg Freiburg Germany; 6 Department of Psychiatry and Psychotherapy Medical Center Albert-Ludwigs-University Freiburg Freiburg Germany; 7 Health Psychology and Applied Biological Psychology Leuphana University Lüneburg Lüneburg Germany; 8 Faculty of Behavioural and Movement Sciences Section of Clinical Psychology Vrije University Amsterdam Amsterdam Netherlands

**Keywords:** pilot project, low back pain, depressive disorder, mental health, sick leave

## Abstract

**Background:**

Chronic back pain (CBP) is linked to a higher prevalence and higher occurrence of major depressive disorder (MDD) and can lead to reduced quality of life. Unfortunately, individuals with both CBP and recurrent MDD are underidentified. Utilizing health care insurance data may provide a possibility to better identify this complex population. In addition, internet- and mobile-based interventions might enhance the availability of existing treatments and provide help to those highly burdened individuals.

**Objective:**

This pilot randomized controlled trial investigated the feasibility of recruitment via the health records of a German health insurance company. The study also examined user satisfaction and effectiveness of a 9-week cognitive behavioral therapy and Web- and mobile-based guided self-help intervention Get.Back in CBP patients with recurrent MDD on sick leave compared with a waitlist control condition.

**Methods:**

Health records from a German health insurance company were used to identify and recruit participants (N=76) via invitation letters. Study outcomes were measured using Web-based self-report assessments at baseline, posttreatment (9 weeks), and a 6-month follow-up. The primary outcome was depressive symptom severity (Center for Epidemiological Studies–Depression); secondary outcomes included anxiety (Hamilton Anxiety and Depression Scale), quality of life (Assessment of Quality of Life), pain-related variables (Oswestry Disability Index, Pain Self-Efficacy Questionnaire, and pain intensity), and negative effects (Inventory for the Assessment of Negative Effects of Psychotherapy).

**Results:**

The total enrollment rate with the recruitment strategy used was 1.26% (76/6000). Participants completed 4.8 modules (SD 2.6, range 0-7) of Get.Back. The overall user satisfaction was favorable (mean Client Satisfaction Questionnaire score=24.5, SD 5.2). Covariance analyses showed a small but statistically significant reduction in depressive symptom severity in the intervention group (n=40) at posttreatment compared with the waitlist control group (n=36; *F*_1,76_=3.62, *P*=.03; *d*=0.28, 95% CI −0.17 to 0.74). Similar findings were noted for the reduction of anxiety symptoms (*F*_1,76_=10.45; *P*=.001; *d*=0.14, 95% CI −0.31 to 0.60) at posttreatment. Other secondary outcomes were nonsignificant (.06≤*P*≤.44). At the 6-month follow-up, the difference between the groups with regard to reduction in depressive symptom severity was no longer statistically significant (*F*_1,76_=1.50, *P*=.11; *d*=0.10, 95% CI −0.34 to 0.46). The between-group difference in anxiety at posttreatment was maintained to follow-up (*F*_1,76_=2.94, *P*=.04; *d*=0.38, 95% CI −0.07 to 0.83). There were no statistically significant differences across groups regarding other secondary outcomes at the 6-month follow-up (.08≤*P*≤.42).

**Conclusions:**

These results suggest that participants with comorbid depression and CBP on sick leave may benefit from internet- and mobile-based interventions, as exemplified with the positive user satisfaction ratings. The recruitment strategy via health insurance letter invitations appeared feasible, but more research is needed to understand how response rates in untreated individuals with CBP and comorbid depression can be increased.

**Trial Registration:**

German Clinical Trials Register DRKS00010820; https://www.drks.de/drks_web/navigate.do? navigationId=trial.HTML&TRIAL_ID=DRKS00010820.

## Introduction

### Background

Chronic back pain (CBP) is a pervasive condition with a 12-month prevalence rate of 38% and a lifetime prevalence of approximately 40% in adults [1]. It is also associated with a 2- to 3-fold increased risk for major depressive disorder (MDD) [2], increased morbidity, and diminished quality of life [3,4]. In addition, depression is a core predictor of persistent pain symptoms, increased pain-related disability, and poor treatment outcomes [5-7]. MDD and CBP each account for 2% of disability-adjusted life years worldwide [8], with immense health care and socioeconomic costs due to productivity losses [9]. Thus, from an individual and societal perspective, it is imperative to provide treatment options that decrease patients’ burden and specifically target individuals’ ability to return to work following sick leave [10].

Effective psychological face-to-face (f2f) treatments exist for depression and CBP [11]. A recent meta-analysis found evidence for the effectiveness of f2f treatments on depression symptoms compared with a nonactive control group (*g*=0.71, 95% CI 0.66 to 0.77) [12]. However, we found no evidence for the effectiveness of multidisciplinary treatments for CBP and comorbid depression. Despite the availability of effective f2f treatments, CBP patients with recurrent depression on sick leave are a difficult-to-reach population with traditional therapy because of a lack of medical and/or disease-related disability specialists.

Internet- and mobile-based interventions (IMIs) have the potential to reach this population because they are easily accessible at any time and in any location. IMIs may be particularly beneficial in psychological and medical treatments as they are accessible and scalable [13]. In addition, the effectiveness of IMIs with mental disorders (eg, depression) [14], disease-related distress in chronic somatic conditions [15], cancer [16], pain [17-19], and coexisting somatic and mental problems (eg, diabetes and depression) [20] is well established.

However, only a few studies have been conducted on the effectiveness of IMIs for individuals with CBP and depression. Recent studies are considering the effectiveness of an IMI on depression in CBP patients following orthopedic rehabilitation, compared with treatment-as-usual (TAU) [21,22]. Irrespective of the findings of these studies, not all patients with CBP seek inpatient rehabilitation treatment. Hence, future research must consider other recruitment strategies. Using health record data might be a valid and innovative recruitment strategy to identify CBP patients with depression.

### Objectives

Thus, one aim of this pilot randomized controlled trial (RCT) was to investigate the feasibility of this recruitment strategy as well as the feasibility, user satisfaction, and effectiveness of a guided IMI for CBP patients with depression on sick leave. The program is conceptualized as a stand-alone intervention to provide help to this difficult-to-reach population and complement conventional health care for CBP patients with depression. We expected the IMI to be more effective in reducing depressive symptom severity and pain-associated measures and in increasing the quality of life compared with a waitlist control condition.

## Methods

### Study Design

This study compares the effectiveness of a guided depression intervention for patients suffering from CBP, resulting in current sick leave, with a waitlist control group (WLC). The intervention was evaluated in a two-armed RCT. The study procedures were approved by the ethical board of the Friedrich-Alexander-University Erlangen-Nürnberg (323_15B), and the trial was registered in the German Clinical Trials Register (DRKS00010820). All study outcomes except for the Structured Clinical Interview (SCID) for the Diagnostic and Statistical Manual of Mental Disorders (DSM-IV) [23] were measured using Web-based self-report assessments at baseline (t1), posttreatment (t2), and a 6-month follow-up (t3). A secure Web-based system (advanced encryption standard, 256-bit encrypted) was used. This study was initially planned with a target sample of 250 participants. However, the trial did not reach the targeted sample of participants (N=76) due to changes in personnel in the insurance company responsible for sending invitation letters. Thus, the planned number of invitation letters to be sent (12,000) was not achieved. The study was initially powered to detect medium effect sizes (*d*=0.40; N=200, power of 95%) and accounted for 25% dropout (N=250). Post hoc analysis with N=76 revealed that we were able to detect an effect size *d* of 0.65 with a power of 80%.

### Procedure

Recruitment was carried out by the study team and supported by a German health insurance company (BARMER) from October 2016 until the end of December 2017 by sending invitation letters to policy holders (N=6000). The inclusion criteria were as follows: (1) recurrent diagnosis of MDD and CBP (M54.x according to ICD-10) [24] in the past 16 months, (2) sick leave for more than a week but less than 6 months, (3) no lifetime diagnosis of psychosis, (4) no nursing care level 2 or higher (eg, needing help at least three or more times a day with body care, food, mobility, and household care), and (5) no acute/recent cancer diagnosis in the past 16 months.

In addition, participants were eligible for the study if they (1) were at least 18 years old, (2) had at least moderate depressive symptoms (Center for Epidemiological Studies Depression Scale; CES-D≥23) [25,26], (3) sufficient German language proficiency, and (4) had access to a computer with internet, an email address, and a mobile phone.

**Figure 1:**
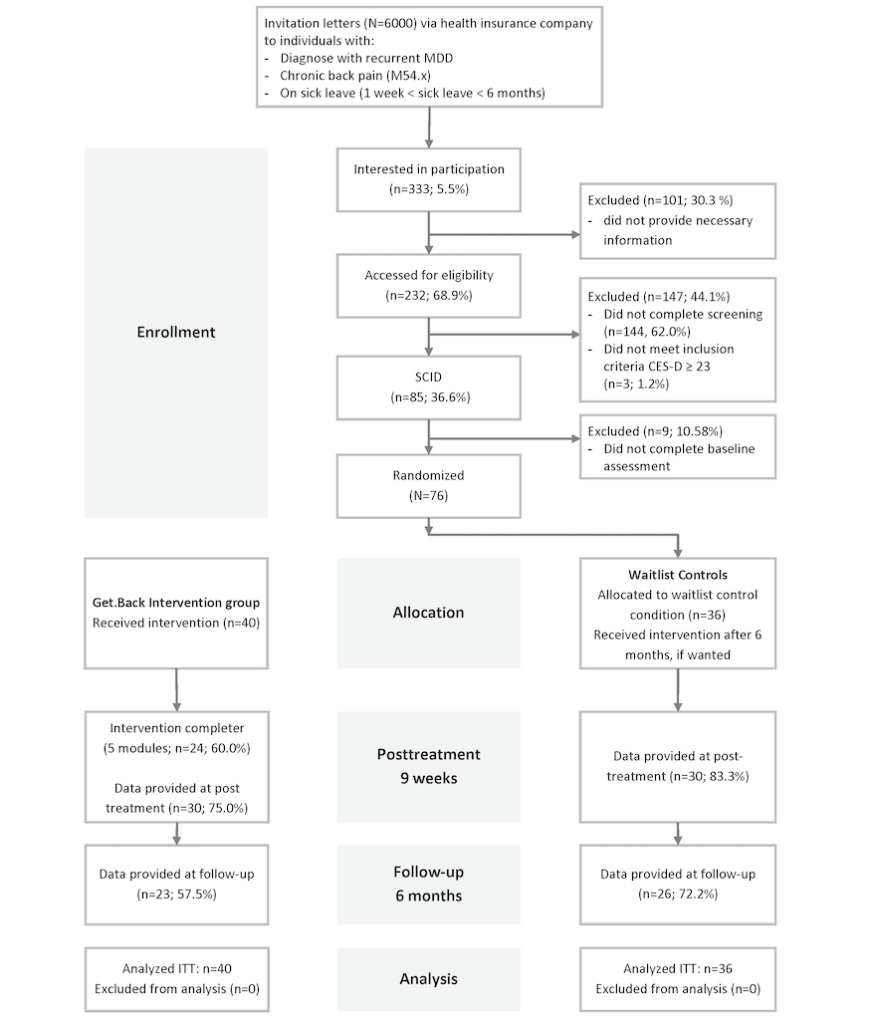
Flowchart for Get.Back. CES-D: Center for Epidemiological Studies Depression; ITT: intention-to-treat; MDD: major depressive disorder; SCID: Structured Clinical Interview for the Diagnostic and Statistical Manual of Mental Disorders; WAI: working alliance.

The exclusion criteria included the following: (1) current psychotherapeutic treatment, (2) exposure to other online trainings provided by the health insurance company, (3) problems with sight or hearing, and (4) a notable suicidal risk indicated by a score greater than 2 on the Beck Depression Inventory–II item number 9 [27,28] and/or suicidal behavior within the last 5 years (assessed during the SCID) [29]. Individuals who were interested in the study contacted the research team and were asked to fill out a brief online screening form to ensure inclusion criteria were fulfilled. After eligible individuals gave the required informed consent for participation, an account for each participant was created. The account’s user name was the participant-provided email address. The account was password protected (password was chosen by participants to prevent misuse of their data). Furthermore, the use of the intervention was free of charge for the study participants. Study procedures are documented in Figure 1.

### Randomization and Blinding

Participants eligible for the study were randomly allocated to one of two groups (intervention group [IG] or WLC) based on an a priori defined list after completing the baseline assessment. An automated, Web-based randomization program [30] was used, which features permuted block randomization. Variable randomly arranged block sizes of 4, 6, 8 and an allocation ratio of 1:1 were adopted. An independent research team member not otherwise involved in the study conducted the randomization. Participants were not blinded to treatment condition.

### Interventions

All participants had unrestricted access to TAU (eg, visiting a general practitioner). Health care utilization data were collected with the well-validated Trimbos and iMTA Questionnaire for costs associated with psychiatric illness (TiC-P; see outcome measures) [31,32].

#### Intervention Group

Participants in the IG had access to the intervention Get.Back. Get.Back is adapted from eSano BackCare-D to suit people on current sick leave [21]. The online intervention is based on cognitive behavioral therapy (CBT) and consists of 7 weekly modules lasting 45 to 60 min each. Modules include information regarding psychoeducation, behavioral activation, problem solving, cognitive restructuring, return to work, self-esteem, and relapse prevention (for a detailed description see Lin et al [21]). eSano BackCare-D was originally adapted from GET.ON Mood Enhancer [33,34]. GET.ON Mood Enhancer was proven to be effective in different populations including individuals with MDD alone [34], individuals with MDD and comorbid diabetes [20], and a subclinically depressed population [35-37]. Get.Back differs from eSano BackCare-D mainly because of content regarding returning to work (for detailed information, see Table 1). In eSano BackCare-D, return to work was included as an optional module, whereas in Get.Back, this module was integrated into the obligatory modules and was extended and improved in content. This module specifically provides stress management strategies (coping with solvable and unsolvable problems in the workplace), psychoeducational information on how to adapt the workplace to each individual’s needs (eg, ergonomic chair and desk arrangement), and relaxation and exercise information to facilitate motion and prevent pain. The return to work module was introduced in the fifth intervention module. The optional modules on partnership, sexuality, and sleep habits from eSano BackCare-D were also used as optional modules in Get.Back. In addition to eSano BackCare-D, we also included 4 optional minimodules (15 min each) on perfectionism, social support, communication, and appreciation that could be completed after module 3, 4, 5, or 6, respectively (for more detailed information, see Table 2). These topics play an important role in acclimating to the workplace after sick leave, and thus, it is crucial to address such information. We also included 1 booster module 4 weeks after the completion of the intervention contrary to 2 booster modules in eSano BackCare-D. The emphasis is on homework assignments, which ideally leads to the application of the learned skills into daily routines. Interactive elements (eg, emails and text messages), reminders, and exercises were used to enhance adherence to the intervention (for detailed information about the intervention, see Multimedia Appendix 1).

**Table 1:** Content of the Get.Back intervention and changes from eSano BackCare-D.

Modules^a^	Depression-specific topics	Back pain–specific topics
1	Psychoeducation	Psychoeducation
2	Behavioral activation	Pain-related complications
3	Problem solving	Problem solving
4	Cognitive restructuring	Pain-related rumination
5	My way back to work: Stress management strategies, psychoeducational information on personal needs at the workplace, relaxation and exercises to facilitate motion and prevent pain, and coping with pain in a work-related environment^b^	My way back to work: Stress management strategies, psychoeducational information on personal needs at the workplace, relaxation and exercises to facilitate motion and prevent pain, and coping with pain in a work-related environment^b^
6	Mood and self esteem^b^ Fostering exercises to value oneself^b^	Strengths and successes despite pain
7	Relapse prevention	Building up and maintaining resources
8	Booster session (within 4 weeks after the regular modules)	Booster session (within 4 weeks after the regular modules)

^a^Original intervention: eSano BackCare-D [21].

^b^Adaptations made to the original intervention content for the Get.Back intervention.

**Table 2:** Content of the optional and minimodules in the Get.Back intervention.

Module		Topics
**Minimodules (15 min)^a^**		On perfectionism, social support, communication, and appreciation
	Perfectionism	Information and exercises on how to cope with perfectionism (especially related to the work environment), cognitive restructuring for a more relaxed and tension free attitude towards tasks
	Social Support	Information and exercises on how to receive and provide social support if needed, interaction in difficult situations (work-related conflicts)
	Communication	Introduction to a concept of nonviolent communication and exercises to facilitate interaction with colleagues and supervisors
	Appreciation	Introduction of mindfulness-based ideas and exercises on how to appreciate positive aspects in daily life routine
Optional modules (45-60 min)		Healthy sleep & intimacy and partnership

^a^Adaptations made to the original intervention content for the Get.Back intervention.

#### Intervention Guidance

Participants were guided by trained psychologists, called eCoaches, who provided semistandardized feedback within 2 working days after each completed module. The feedback was based on an eCoach manual, which is intended to ensure adherence to the treatment. The manual also includes instructions to remind, set deadlines, and formulate standardized feedback. The communication between eCoaches and participants occurred through Get.Back’s online platform. The feedback content was based on the participant’s statements and included positive reinforcement to encourage participants to continue with the training. If any further questions arose, participants and eCoaches were able to contact each other at any time via the platform. In case of noncompletion of the modules, eCoaches sent reminders to participants. eCoaches received a training based on the eCoach manual and on previous experiences by the trainers as well as constant supervision during their time as eCoaches on this study. Training and supervision were provided by a trained and fully licensed (according to German laws and regulations) behavioral and cognitive psychotherapist.

#### Text Message Coach

Participants had the option to receive daily standardized text messages to increase treatment outcomes and adherence, as well as to support transferring learned skills into their daily routine. Content included the following: (1) reminders to complete weekly assignments, (2) repetition of the content, and (3) motivation enhancement components. Each participant received a total of 42 text messages.

#### Waitlist Control Group

Participants in the WLC had access to the unguided intervention after study completion in addition to unrestricted access to TAU throughout their participation.

### Outcomes

#### Primary Outcome - Depressive Symptom Severity at Posttreatment

Depressive symptom severity was measured with CES-D [25,26], a widely used instrument in IMI depression trials [14,38]. The 20 items refer to the previous week and are answered on a 4-point Likert scale, ranging from 0 (*rarely or none of the time*) to 3 (*most or all of the time*) with a total score ranging from 0 to 60. Items include the most common symptoms related to depression, such as low mood, loss of appetite, concentration difficulties, and hopelessness. CES-D scores of 16 or greater indicate clinically relevant levels of depression severity. The CES-D has been shown to have excellent reliability (ie, internal consistency of Cronbach alpha=.89) [39]. In this study, Cronbach alpha was .82.

#### Secondary Outcomes

##### Depression Symptoms

Quick Inventory of Depressive Symptomatology Self-Report (QIDS-SR16) [40,41] is a 16-item questionnaire that assesses all criteria for MDD according to DSM-5 [42]. The items refer to the previous week and are answered on a 4-point Likert scale, ranging from 0 (absence of symptom for 7 days) to 3 (presence of intense symptoms every day). Total scores range from 0 to 27, with the following cutoffs: 0 to 5 indicates no depression, 6 to 10 indicates mild depression, 11 to 15 indicates moderate depression, 16 to 20 indicates severe depression, and 21 to 27 indicates very severe depression. Psychometric properties are reported to be adequate (Cronbach alpha was .77) [43]. In this study, Cronbach alpha was .74.

##### Quality of Life

Assessment of Quality of Life (AQoL-6D) [44] was used to measure the health-related quality of life. AQoL-6D includes 20 items and covers 6 dimensions. Psychometric properties of AQoL-6D are well established [44]. In this study, Cronbach alpha was .85. We also used EuroQoL [45,46], a widely implemented instrument, which covers 5 health domains. In this study, Cronbach alpha was .76.

##### Anxiety

The Hamilton Anxiety and Depression Scale [47,48] is a 7-item self-report measure that assesses anxiety and depressive symptoms during the last 7 days on two subscales. In this study, only the anxiety subscale was used. Items are answered on a 4-point Likert scale with total scores ranging from 0 to 21 on the anxiety subscale. Cutoffs are as follows: 8 to 10 indicates mild anxiety, 11 to 14 indicates moderate anxiety, and 15 to 21 indicates severe anxiety. Psychometric properties are reported to be adequate [47]. Cronbach alpha in this study was .70.

##### Pain-Related Disability

The Oswestry Disability Index [49,50] is a 10-item self-report questionnaire with good validity and reliability [51]. Total scores can also be used to calculate a correlated functional disability at the individual level (measured in percentages, ranging from 0% to 100%). Cutoffs are as follows: 0% to 20% indicates minimal disability, 21% to 40% indicates moderate disability, 41% to 60% indicates severe disability, 61% to 80% indicates crippled, and 81% to 100% indicates individuals who are either bedbound or exaggerating their symptoms [49,52]. In this study, Cronbach alpha was .90.

##### Pain Rating

We used three items measured on an 11-point numerical (0-10) scale regarding the worst, least, and average pain during the last week. The three items were averaged to calculate a global pain rating over the past week. In addition, we assessed pain using a categorical rating of pain intensity (none, mild, moderate, and severe).

##### Pain-Related Self-Efficacy

The Pain Self-Efficacy Questionnaire [53,54] is a valid and reliable 10-item instrument that assesses self-efficacy expectations related to pain on a 7-point Likert scale. Total scores range from 0 to 60, and higher scores represent more self-efficacy. In this study, Cronbach alpha was .89.

##### Screening for Bipolar Disorder

The Mood Disorder Questionnaire (MDQ) [55] is a brief self-report instrument which comprises three sections. In the first section, 13 manic and hypomanic symptoms are assessed on a dichotomous scale (ie, yes and no). Section two asks if any of these symptoms are experienced at the same time, which is also answered on a dichotomous scale. Section three is answered on a 4-point Likert scale (no problems to serious problems) regarding the degree to which their symptoms have caused problems. The screening is considered positive if a cutoff of ≥7 symptoms for section one, *yes* in section two, and a problem severity of *moderate* or *serious* in section three are indicated. Psychometric properties are well validated, with a reported sensitivity of 0.28 and a specificity of 0.97 [56].

##### Working Capacity

The Subjective Prognostic Employment Scale [57] is a 3-item self-report questionnaire, with a sum score from 0 to 3. It is well validated (internal consistency according to Guttman scale: rep=0.99) [57]. The rep=0.99 refers to the coefficient of reproducibility and can be considered as a measure of internal consistency. The coefficient ranges from 0 (no reproducibility of data) to 1 (perfect reproducibility of data) with values of 0.90 and above indicating acceptable reproducibility.

##### Client Satisfaction

The Client Satisfaction Questionnaire (CSQ) [58,59] adapted for the assessment of client satisfaction in IMIs by Boß et al [60] consists of 8 items that are rated on 4-point and 5-point Likert scales. CSQ was only assessed in the IG. In this study, Cronbach alpha was .93.

##### Adverse Events

The Inventory for the Assessment of Negative Effects of Psychotherapy (INEP) [61] was used to assess negative effects during posttreatment online assessments. The 15-item INEP assesses common changes participants may have experienced in line with the intervention’s 5 domains (intrapersonal change, relationship, friends and family, work, and stigma). This study had a Cronbach alpha of .55. To further assess serious adverse events (SAE), participants were asked about adverse events at the beginning of each module and were also encouraged to report any such events to their eCoach who monitored the SAEs and initiated further actions if needed. Symptom deterioration was assessed by calculating the reliable change index [62] for CES-D (for a detailed description, see Statistical Analysis).

##### Working Alliance

To evaluate a subjective rating of the alliance between eCoach and patient, the short, revised version of the working alliance (WAI) [63,64] was administered only after the third module (half of the intervention). The WAI–short revised is a well validated [65], 12-item questionnaire and consists of three subscales assessing (1) how closely the client and therapist agree on and are mutually engaged in the goals of treatment (task subscale); (2) how closely the client and therapist agree on how to reach the treatment goals (goal subscale); and (3) the degree of mutual trust, acceptance, and confidence between the client and therapist (bond subscale). Items are rated on a 5-point Likert scale from 1 (*seldom*) to 5 (*always*). Cronbach alpha in this study was .92.

##### Health Care Utilization and Sick Leave Data

Health care utilization data and data on sick leave were collected with the well-validated TiC-P illness [31,32] via online self-report.

##### Adherence

The attrition rate was calculated by identifying the percentage of individuals who no longer utilized the intervention, as indicated in their log-in data. This provides an estimate of the participants’ intervention adherence.

### Statistical Analysis

All analyses were conducted using SPSS Statistics Version 25 (IBM Corporation) [66] and are reported in accordance with the Consolidated Standards of Reporting Trials statement [67]. Missing data were multiply imputed using a Markov chain Monte Carlo [68] multivariate imputation algorithm with 50 estimations per missing value in accordance with the intention-to-treat principle. Descriptive statistics were reported for feasibility of recruitment, intervention usage, client satisfaction, and relationship with the eCoach. Analyses of covariance adjusted for sex, age, and baseline symptom severity were performed to analyze primary and secondary outcomes between groups at posttreatment and the 6-month follow-up. In a sensitivity analysis, the same analyses were performed with the last observation carried forward (LOCF) method for the postassessment and follow-up. In addition, we performed per-protocol analyses to assess differences in the primary outcome between intervention completers and noncompleters. Participants were classified as intervention completers if they adhered to at least 80% of the intervention (5 out of 7 modules).

Results are reported as mean within- and between-group differences and as Cohen *d* effect sizes (and their 95% CIs, according to Hedges and Olkin [69]) controlling for baseline scores (ie, calculating change scores divided by the pooled standard deviation of change scores). To assess improvements in the primary outcome (depressive symptom severity) at the individual level, treatment response and near-to-symptom-free status (eg, CES-D<16) were calculated at posttreatment and the 6-month follow-up. In addition, corresponding numbers needed to treat (NNT, with 95% CI) to achieve symptom-free status were calculated at posttreatment and the 6-month follow-up. Treatment response was defined as a 50% symptom reduction from baseline to follow-up, as well as based on the reliable change index by Jacobson and Truax [62]. Participants with a reliable positive change in depression (RCI>1.96; CES-D≥ −12.10; CES-D points take into account the reliability of the CES-D to compensate for measurement errors) were classified as responders to the intervention. Accordingly, symptom deterioration was classified as an increase in 7.8 CES-D points between baseline and posttreatment assessments, and between baseline and the 6-month follow-up. Statistical significance in all analyses was set at alpha<.05 and was one-sided according to Cho and Abe [70].

## Results

### Descriptive Statistics

In total, 76 participants were included in the study. For detailed information on characteristics, see Table 3. There were no clinically relevant differences in baseline characteristics between the groups.

The posttreatment (9-week) questionnaire return rate was 79% (60/76). Of those, 75% (30/40) of participants were in the IG and 83% (30/36) of participants were in the WLC. Complete data at the 6-month follow-up were collected from 58% (23/40) of participants in the IG and 72% (26/36) of participants in the WLC, with an overall completion rate of 64% (49/76). Dropout rates did not statistically differ at posttreatment (Χ^2^_1_=0.7, *P*=.37) or at the 6-month follow-up (Χ^2^_1_=1.7, *P*=.18). Participants in the study were predominately female with an average age of 50.78 years (SD 7.85). The majority of participants had a midlevel of education (equivalent to General Educational Development Test) and were married. The average age at depression onset was 35.19 years (SD 14.64), and the average number of previous depressive episodes was 8.2 (SD 7.27). Self-reported depressive symptom severity measured with CES-D was 32.92 (SD 7.52). The most common depressive episode severity (QIDS) was moderate (24/76, 32%) or severe (28/76, 37%). In total, 9% (7/76) of participants screened positive for bipolar disorder (MDQ). The pain-related disability (ODI) was 27.3%, which corresponds to a moderate disability. The average pain intensity was 4.39 (SD 1.94, range 0-11), corresponding to a moderate level of pain present during the last week. The most common categorical rating on the actual pain intensity was moderate (39/76, 51%).

**Table 3:** Demographics and clinical characteristics.

Variable		IG^a^ (n=40)	WLC^b^ (n=36)	Total (N=76)
Age (years), mean (SD)		51.3 (8.60)	50.1 (7.00)	50.78 (7.85)
Sex, female, n (%)		26 (65)	29 (81)	55 (72)
**Education, n (%)**				
	High	8 (20)	9 (25)	17 (22)
	Medium	26 (65)	25 (69)	51 (67)
	Low	6 (15)	2 (6)	8 (11)
**Marital status^c^, n (%)**				
	Single/separated	8 (20)	14 (39)	22 (29)
	Married/in a relationship	31 (78)	21 (58)	52 (68)
	Widowed	1 (3)	1 (3)	2 (3)
Number of depressive episodes, mean (SD)		7.85 (6.39)	8.60 (8.26)	8.20 (7.27)
Age at onset (years), mean (SD)		36.4 (14.4)	33.8 (14.9)	35.19 (14.6)
**Severity of current episode^d^, n (%)**				
	Mild	9 (23)	5 (14)	14 (18)
	Moderate	12 (30)	12 (33)	24 (32)
	Severe	15 (38)	13 (36)	28 (37)
	Very severe	4 (10)	6 (17)	10 (13)
**Current pain intensity, n (%)**				
	None	3 (8)	4 (11)	7 (9)
	Mild	15 (38)	12 (33)	27 (36)
	Moderate	20 (50)	19 (53)	39 (51)
	Severe	2 (5)	1 (3)	3 (4)
**Social support^c^, n (%)**				
	High	10 (25)	10 (28)	20 (26)
	Medium	12 (30)	14 (39)	26 (34)
	Low	18 (45)	12 (33)	30 (39)
Partial disability, yes, n (%)		0 (0)	3 (8)	3 (4)
Positive screening for bipolar disorder, yes, n (%)		4 (10)	3 (8)	7 (9)

^a^IG: intervention group.

^b^WLC: waitlist control group.

^c^Percentages less than 100 are due to missing data.

^d^Measured with Quick Inventory of Depressive Symptomatology.

### Use of Other Health Care Services and Sick Leave Change

Data on concurrent mental health care service use was provided by 58% (44/76) of participants at the 6-month follow-up (IG: 21/44, 48%; WLC: 23/44, 52%). In total, 84% (37/44) of participants reported visits to their GP in the previous 3 months, with more participants (21/23, 91%) in the WLC compared with the IG (16/21, 76%; Χ^2^_1_=1.8, *P*=.17). About one-third of participants reported visits to a psychotherapist and/or a specialist in neurology and psychiatry, with no notable differences between study groups (15/44, 34%; IG: 7/21, 33%; WLC: 8/23, 35%; Χ^2^_1_=0.0, *P*=.83). Approximately two-thirds of participants (26/40, 65%), with an equal number of participants in the IG (13/26, 50%) and WLC (13/26, 50%), used pain management medication for back pain with no difference between the groups (Χ^2^_1_=0.0, *P>*.99). Half of the participants (23/39, 59%) took antidepressant medication (IG: 12/23, 52%; WLC: 11/23, 48%) with no statistical difference between the groups (Χ^2^_1_=0.2, *P*=.60). Data on current sick leave were provided by 50% of participants (IG: 16/40, WLC: 22/36). At the 6-month follow-up, 33% (25/76) of study participants reported being on sick leave during the last 3 months. There were more participants in the WLC (n=17/22) reporting to have been on sick leave than in the IG (n=8/16). However, there was no statistically significant difference between the groups (Χ^2^_1_=3.0, *P*=.08).

### Feasibility: Feasibility of Recruitment, Intervention Usage, Client Satisfaction, and Relationship With the eCoach

#### Feasibility of Recruitment

Of the 6000 individuals who were sent invitations, interest in the study was expressed by 333 (5.50%) individuals. However, only 3.86% (232/6000) of individuals started the screening process. Of those 232 individuals, 144 (62.0%) did not complete the screening, while 3 participants (1.2%) did not meet the inclusion criteria. In total, 36.6% (85/232) of the screened individuals were eligible for study participation and were invited for a diagnostic interview via telephone (see Figure 1). Of these 85 individuals, 9 (11%) did not complete the baseline assessment after the telephone interview and were excluded, resulting in 76 (N) study participants. In total, the enrollment rate of those who received invitation letters was 1.26% (76/6000).

In terms of costs, the total cost of recruitment was 2683.20€ (US $2974.13), and corresponding costs of approximately 8.05€ (US $8.92) per individual signing up for participation. The cost associated with every finally enrolled individual (ie, intervention implementation costs) was 35.30€ (US $39.13) per person.

#### Intervention Usage

Participants completed on average 4.8 (SD 2.6) modules of the intervention. In total, 60% (24/40) of participants in the IG were identified as completers, and 55% (22/40) of participants adhered to all 7 modules. Of the 16 (16/40, 40%) participants who did not complete at least 5 modules, 1 (3%) participant never started the intervention. Completers and noncompleters did not differ in their baseline characteristics.

#### Client Satisfaction

Participants were generally satisfied with the intervention. The average score on the CSQ-8 was 24.53 (SD 5.20, range 8-32, min=10, max=32). A high quality and satisfaction rating of the intervention was reported by 90% (26/29) of participants, who stated that they would recommend the intervention to a friend. The vast majority of participants (25/29, 86%) stated that they would use the intervention again if the need arose. Four-fifths of the participants received the training that they wanted (24/29, 83%), perceived the intervention as helpful in dealing with their problems more effectively, and were overall satisfied with the treatment (23/29, 79%). Three-quarters of participants (22/29) also reported that the intervention met their needs and that they were satisfied with the amount of help they received throughout the intervention.

#### Relationship With the eCoach

Analysis of WAI-SR showed a good WAI between participants and eCoaches with a mean score of 39.30 (SD 11.64, range 15-60, min=21, max=56). Participant’s ratings of the subscales revealed the highest ratings in the subscale task (mean 14.22, SD 3.77, range 5-20, min=8, max=20), followed by the goal subscale (mean 13.94, SD 3.70, range 5-20, min=8, max=20) and the bond subscale (mean 11.13, SD 4.98, range 5-20, min=4, max=19).

### Short-Term Effects

#### Primary Intervention Outcome

The mean scores for outcomes are reported in Table 4. Table 5 displays results for all outcome measures. The results revealed statistically significant reductions in the primary outcome from baseline to posttreatment in both the IG (reduction of 6.84 points on CES-D; t_40_=5.82, *P*<.001; *d*=0.84, 95% CI 0.39 to 1.30) and WLC (reduction of 4.64 points on CES-D; t_36_=3.86, *P*<.001; *d*=0.64, 95% CI 0.17 to 1.12). There was a statistically significant difference between the IG and WLC at posttreatment, resulting in a small between-group effect size favoring the intervention condition (*F*_1,76_=3.62, *P*=.03; *d*=0.28, 95% CI −0.17 to 0.74). There were no significant differences in the primary outcome between intervention completers and noncompleters (*F*_1,29_=0.01; *P=*.97).

**Table 4:** Mean (SD) of outcomes.

Outcomes	Baseline			Posttreatment			6-month follow-up		
	IG^a^ (n=40), mean (SD)	WLC^b^ (n=36), mean (SD)	Total (N=76), mean (SD)	IG (n=40), mean (SD)	WLC (n=36), mean (SD)	Total (N=76), mean (SD)	IG (n=40), mean (SD)	WLC (n=36), mean (SD)	Total (N=76), mean (SD)
CES-D^c^	32.50 (7.27)	33.55 (7.84)	32.92 (7.52)	25.66 (8.48)	28.91 (6.38)	27.20 (7.68)	24.36 (9.03)	26.40 (7.13)	25.37 (8.20)
QIDS^d^	15.00 (4.57)	15.55 (4.53)	15.62 (4.53)	13.06 (4.35)	14.21 (3.15)	13.60 (3.85)	12.76 (4.32)	14.25 (3.54)	13.46 (4.08)
AQoL-6D^e^	0.48 (0.16)	0.47 (0.16)	0.48 (0.16)	0.55 (0.17)	0.51 (0.14)	0.53 (0.15)	0.60 (0.18)	0.55 (0.12)	0.57 (0.16)
EQ-5D-5L^f^	0.64 (0.21)	0.66 (0.18)	0.65 (0.19)	0.67 (0.19)	0.68 (0.17)	0.67 (0.18)	0.69 (0.17)	0.68 (0.15)	0.68 (0.16)
HADS^g^-anxiety	12.18 (3.47)	11.80 (3.30)	12.00 (3.37)	9.34 (3.43)	11.20 (3.11)	10.22 (3.39)	8.57 (3.21)	9.56 (3.21)	9.04 (3.23)
ODI-fd^h^	28.50 (17.97)	26.11 (16.79)	27.39 (17.35)	28.26 (16.29)	25.56 (16.52)	26.98 (16.53)	25.15 (13.43)	24.90 (15.27)	25.03 (14.23)
Average pain intensity	4.68 (1.94)	4.08 (1.91)	4.39 (1.94)	4.68 (1.86)	3.81 (1.76)	4.27 (1.85)	3.89 (1.60)	3.67 (1.80)	3.79 (1.69)
PSEQ^i^	33.72 (12.30)	34.75 (11.50)	34.21 (11.86)	36.67 (11.87)	36.62 (9.38)	36.65 (10.69)	40.14 (13.42)	38.20 (9.70)	39.22 (11.77)
SPE^j^	0.95 (0.74)	0.99 (0.82)	0.97 (0.78)	1.03 (0.66)	0.9 (0.76)	0.97 (0.71)	1.69 (0.34)	1.6 (0.42)	1.65 (0.38)

^a^IG: intervention group.

^b^WLC: waitlist control group.

^c^CES-D: Center of Epidemiological Studies Depression Scale.

^d^QIDS: Quick Inventory of Depressive Symptomatology.

^e^AQoL-6D: Assessment of Quality of life.

^f^EQ-5D-5L: EuroQol.

^g^HADS: Hamilton Anxiety and Depression Scale.

^h^ODI-fd: Oswestry Disability Index-functional disability, measured as % (SD).

^i^PSEQ: Pain Self-Efficacy Questionnaire.

^j^SPE: Subjective Prognosis of Employment Scale.

**Table 5:** Results of all outcomes.

Time points and outcomes			*F* test (*df*)	*P* value	Between-group		IG^a^		WLC^b^	
					*d*	95% CI	*d*	95% CI	*d*	95% CI
**Posttreatment**										
	**Primary outcome**									
		CES-D^c^	3.62 (1,76)	0.03	0.28	−0.17 to 0.74	0.86	0.39 to 1.30	0.64	0.17 to 1.12
	**Secondary outcomes**									
		QIDS^d^	1.24 (1,76)	0.13	0.16	−0.28 to 0.62	0.43	0.01 to 0.88	0.34	−0.12 to 0.82
		AQoL-6D^e^	0.99 (1,76)	0.16	0.2	−0.24 to 0.66	0.39	−0.04 to 0.84	0.26	−0.19 to 0.73
		EQ-5D-5L^f^	0.01 (1,76)	0.44	0.07	−0.37 to 0.52	0.14	−0.29 to 0.58	0.09	−0.36 to 0.55
		HADS^g^-anxiety	10.45 (1,76)	0.001	0.14	−0.30 to 0.60	0.81	0.36 to 1.27	0.18	−0.27 to 0.65
		ODI^h^	0.15 (1,76)	0.35	0.02	−0.42 to 0.47	0.01	−0.42 to 0.45	0.03	−0.42 to 0.49
		Average pain intensity	3.76 (1,76)	0.06	0.23	−0.22 to 0.68	0	−0.44 to 0.43	0.14	−0.32 to 0.60
		PSEQ^i^	0.02 (1,76)	0.43	0.11	−0.33 to 0.56	0.24	−0.19 to 0.68	0.17	−0.28 to 0.64
		SPE^j^	1.35 (1,76)	0.12	0.24	−0.20 to 0.70	0.11	−0.32 to 0.56	0.11	−0.34 to 0.58
**6-month follow-up**										
	**Primary outcome**									
		CES-D	1.50 (1,76)	0.11	0.1	−0.34 to 0.46	0.98	0.51 to 1.46	0.94	0.45 to 1.43
	**Secondary outcomes**									
		QIDS	1.93 (1,76)	0.08	0.23	−0.21 to 0.69	0.5	0.06 to 0.95	0.32	−0.14 to 0.79
		AQoL-6D	1.44 (1,76)	0.11	0.21	−0.23 to 0.66	0.65	0.20 to 1.10	0.55	0.08 to 1.02
		EQ-5D-5L	0.06 (1,76)	0.38	0.16	−0.29 to 0.61	0.22	−0.21 to 0.66	0.08	−0.37 to 0.54
		HADS-anxiety	2.94 (1,76)	0.04	0.38	−0.07 to 0.83	1.07	0.60 to 1.54	0.68	0.21 to 1.16
		ODI	0.11 (1,76)	0.36	0.14	−0.30 to 0.59	0.21	−0.22 to 0.65	0.07	−0.38 to 0.53
		Average pain intensity	0.03 (1,76)	0.42	0.21	−0.24 to 0.66	0.44	0.00 to 0.88	0.22	−0.24 to 0.68
		PSEQ	0.57 (1,76)	0.22	0.23	−0.21 to 0.68	0.39	−0.04 to 0.83	0.51	0.04 to 0.98
		SPE	0.96 (1,76)	0.16	0.15	−0.29 to 0.61	−1.27	0.79 to 1.76	−0.91	0.43 to 1.41

^a^IG: intervention group.

^b^WLC: waitlist control group.

^c^CES-D: Center of Epidemiological Studies Depression Scale.

^d^QIDS: Quick Inventory of Depressive Symptomatology.

^e^AQoL-6D: Assessment of Quality of Life.

^f^EQ-5D-5L: EuroQol.

^g^HADS: Hamilton Anxiety and Depression Scale.

^h^ODI: Oswestry Disability Index.

^i^PSEQ: Pain Self-Efficacy Questionnaire.

^j^SPE: Subjective Prognosis of Employment Scale.

#### Treatment Response

Reliable change did not significantly differ between participants in the IG (17/40, 43%) and WLC (11/40, 31%; Χ^2^_1_=1.1, *P=*.14; NNT=8, 95% CI 3 to 10^6^). A nonsignificant score reduction of 50% from baseline to posttreatment was seen more often in the IG (2/77, 3%) compared with the WLC (n=0; Χ^2^_1_=1.8, *P=*.08; NNT=20, 95% CI 9 to 10^6^).

#### Near-to-Symptom-Free Status

Significantly more participants in the IG (5/40, 13%) reached a symptom-free status compared with the WLC (n=0; Χ^2^_1_=4.8, *P=.*01*;* NNT=8, 95% CI 5 to 45).

#### Secondary Outcomes

The IG showed a significantly greater reduction in anxiety compared with the WLC (*F*_1,76_=10.45, *P*=.001; *d*=0.14, 95% CI −0.31 to 0.60) with a within-group effect size *d* of 0.81 (95% CI 0.36 to 1.28; t_40_=5.40; *P*<.001) versus 0.18 (95% CI −0.27 to 0.65; t_36_=1.26; *P*=.21) in the WLC. There were no statistically significant differences between the IG and WLC with regard to any other secondary outcomes (eg, pain-related disability, self-reported depressive symptoms, pain-related self-efficacy, quality of life, and subjective prognosis of employment; see Table 5).

#### Adverse Events

At posttreatment, 10% (4/40) of participants reported at least 1 negative event related to the intervention. In total, 6 negative events were reported by the IG, with the most commonly reported negative event being: “Since the start of the intervention, I suffer more from events in the past” (n=3). In addition, 17% (5/30) of participants reported at least 1 negative event not related to the training. Symptom deterioration did not take place in the IG. In the WLC, 3% (1/37) of participants did experience deterioration. This difference was not statistically significant (Χ*^2^*_1_=1.1; *P=*.14).

### Long-Term Effects

#### Primary Intervention Outcome

Both study groups displayed statistically significant reductions in depressive symptom severity from baseline to the 6-month follow-up (IG: t_40_=5.99, *P*<.001; *d*=0.98; 95% CI 0.51 to 1.46 and WLC: t_36_=4.99, *P*<.001; *d*=0.94; 95% CI 0.43 to 1.45); however, the between-group difference was not statistically significant (*F*_1,76_=1.50, *P*=.11; *d*=0.10, 95% CI −0.34 to 0.46).

#### Treatment Response, Near-to-Symptom-Free Status, and Symptom Deterioration

A reliable change from baseline to the 6-month follow-up was more often seen in the IG (9/40, 23%) compared with the WLC (6/36, 17%). However, this difference was not statistically significant (Χ^2^_1_=0.4, *P=*.52; NNT=17, 95% CI 5 to 10^6^). A symptom reduction of 50% from baseline to follow-up was seen in twice as many participants in the IG (6/40, 15%) compared with the WLC (3/36, 8%), but this difference was not statistically significant (Χ^2^_1_=0.8; *P=*.18). In all, 48% (19/40) of participants in the IG and 39% (14/36) of participants in the WLC reached symptom-free status at the 6-month follow-up, with no statistically significant difference between the groups (Χ^2^_1_=0.5; *P=*.22). From baseline to follow-up, symptom deterioration occurred more often in the WLC, with 6% (2/36) of participants, compared with 3% (2/40) of participants in the IG; however, this difference was not statistically significant (Χ*^2^*_1_=0.4; *P=*.24).

#### Secondary Outcomes

Analyses revealed that the between-group difference in anxiety at posttreatment was also statistically significant at follow-up (*F*_1,76_=2.94, *P*=.047; *d*=0.38, 95% CI −0.07 to 0.83). There were no statistically significant differences across groups with regard to any other secondary outcomes (eg, pain-related disability, self-rated depressive symptoms, average pain intensity, pain-related self-efficacy, quality of life, or subjective prognosis of employment; Table 5).

### Sensitivity Analysis

Results of the sensitivity analyses were similar to the results of the main analyses. It has previously been shown that LOCF estimates similar effect sizes, but overestimates the precision, compared with multiple imputation [71]. Results of the sensitivity analyses are presented in Table 6.

**Table 6:** Sensitivity analyses (last observation carried forward).

Time points and outcomes			*F* test (*df*)	*P* value	Between-group		IG^a^ (n=40)		WLC^b^ (n=36)	
					*d*	95% CI	*d*	95% CI	*d*	95% CI
**Posttreatment**										
	**Primary outcome**									
		CES-D^c^	3.64 (1,76)	0.03	0.34	−0.11 to 0.80	0.61	0.16 to 1.06	0.34	−0.12 to 0.81
	**Secondary outcomes**									
		QIDS^d^	3.79 (1,76)	0.02	0.37	−0.08 to 0.83	0.39	−0.04 to 0.84	0.17	−0.29 to 0.64
		AQoL-6D^e^	2.60 (1,76)	0.05	0.37	−0.08 to 0.82	0.34	−0.10 to 0.78	0.11	−0.35 to 0.58
		EQ-5D-5L^f^	1.94 (1,76)	0.08	0.35	−0.10 to 0.81	0.23	−0.20 to 0.68	0	−0.46 to 0.46
		HADS-anxiety^g^	9.34 (1,76)	0	0.75	0.28 to 1.22	0.62	0.18 to 1.08	0.07	−0.39 to 0.54
		ODI^h^	2.38 (1,76)	0.06	0.37	−0.08 to 0.83	0.11	−0.32 to 0.56	0.05	−0.41 to 0.52
		Average pain intensity	1.24 (1,76)	0.13	0.08	−0.37 to 0.53	0.04	−0.39 to 0.48	0.08	−0.37 to 0.55
		PSEQ^i^	3.62 (1,76)	0.03	0.46	0.01 to 0.92	0.23	−0.21 to 0.67	0.04	−0.42 to 0.50
		SPE^j^	0.25 (1.76)	0.3	0.13	−0.32 to 0.58	0.1	−0.34 to 0.54	0.03	−0.43 to 0.49
**6-month follow-up**										
	**Primary outcome**									
		CES-D	3.35 (1,76)	0.04	0.31	−0.14 to 0.77	0.69	0.24 to 1.14	0.4	−0.06 to 0.87
	**Secondary outcomes**									
		QIDS	3.81 (1,76)	0.02	0.42	−0.03 to 0.88	0.44	0.00 to 0.89	0.16	−0.30 to 0.63
		AQoL-6D	2.36 (1,76)	0.06	0.34	−0.11 to 0.80	0.55	0.10 to 1.00	0.31	−0.15 to 0.78
		EQ-5D-5L	2.56 (1,76)	0.06	0.44	−0.01 to 0.90	0.37	−0.07 to 0.82	0	−0.46 to 0.47
		HADS-anxiety	3.95 (1,76)	0.03	0.51	0.05 to 0.97	0.71	0.26 to 1.17	0.31	−0.15 to 0.78
		ODI	4.89 (1,76)	0.02	0.53	0.08 to 0.99	0.26	−0.18 to 0.70	0.08	−0.38 to 0.55
		Average pain intensity	0.73 (1,76)	0.19	0.3	−0.15 to 0.75	0.32	−0.12 to 0.77	0.09	−0.36 to 0.56
		PSEQ	3.13 (1,76)	0.04	0.42	−0.03 to 0.88	0.4	−0.04 to 0.84	0.03	−0.42 to 0.50
		SPE	0.00 (1,76)	0.98	0.03	−0.41 to 0.49	0.63	0.18 to 1.08	0.58	0.11 to 1.05

^a^IG: intervention group.

^b^WLC: waitlist control group.

^c^CES-D: Center of Epidemiological Studies Depression Scale.

^d^QIDS: Quick Inventory of Depressive Symptomatology.

^e^AQoL-6D: Assessment of Quality of Life.

^f^EQ-5D-5L: EuroQol.

^g^HADS: Hamilton Anxiety and Depression Scale.

^h^ODI: Oswestry Disability Index.

^i^PSEQ: Pain Self-Efficacy Questionnaire.

^j^SPE: subjective Prognosis of Employment Scale.

## Discussion

### Principal Findings

Delivery of CBT via the internet seems feasible in a highly burdened sample, and the enrollment rate was 1.26% (76/6000). As hypothesized, Get.Back demonstrated small but statistically significant effects compared with the WLC in terms of reducing depressive symptom severity at posttreatment. However, findings did not support effectiveness with regard to pain measures, quality of life, or long-term effectiveness.

### Comparison With Previous Research

To the best of our knowledge, there are no published studies regarding digital or f2f psychological interventions for patients with comorbid depression and CBP on sick leave. Our findings regarding the feasibility and user satisfaction of IMIs are in line with other studies for monodisorder depression and for comorbid depression with somatic diseases [19,72].

The within-group effect size *d* of 0.86 in favor of the IG is comparable with existing evidence for digital interventions for MDD. Königbauer et al [14] found standardized within-group effect sizes (Hedge *g*) ranging from −0.64 (95% CI −1.27 to −0.01) to −1.52 (95% CI −2.22 to −0.82) for the reduction in depressive symptom severity at posttreatment. However, between-group effect sizes found in this study were smaller than those in similar trials on IMIs with depressed individuals. One reason might be the notable improvements in the WLC. Participants in the WLC knew that they were scheduled to get access to Get.Back after a waiting period. Therefore, there is a possibility of an expectancy effect. A recent study showed that patients with MDD who were scheduled to wait for treatment showed a significant decline in depressive symptoms [73]. However, WLCs may also experience a nocebo effect, such that participation as a waitlist control might reduce natural recovery [74]. Future studies are needed to better understand the effects of scheduled waiting in clinical trials.

Moreover, regardless of the treatment format, psychological interventions for depression in individuals with CBP might achieve lower effects compared with individuals without CBP. This hypothesis is supported by a meta-analysis showing that depression treatments tend to be less effective in individuals with general medical disorders compared with nonmedical populations [12].

There is existing evidence that CBT is effective in reducing depressive symptom severity in individuals with CBP [21]. However, this evidence is limited to samples with unspecified depression diagnoses and symptoms. Moreover, internet-based self-help could be less suitable for this group compared with f2f psychotherapy. To date, there is no RCT in an f2f setting that investigates the effectiveness of a CBT depression intervention in individuals with CBP, and only 1 other trial that investigates iCBT in individuals with CBP and clinical depression [21]. We found no trials focusing on individuals with chronic depression or current sick leave.

Furthermore, this study aimed to reach individuals who are not actively seeking help, meaning that their motivation to change might be lower compared with individuals who do actively seek help. In Germany, the decision to pursue psychological treatments for mental illness is made after an average waiting period of approximately 7 years [75]. Therefore, motivation for change can be considered an important predictor in depression treatments and related outcomes [76]. However, it could be that Get.Back combined with our recruitment strategy resulted in smaller effects compared with recruitment strategies directly targeting individuals who are actively seeking help. Thus, it is possible that this intervention may have increased effects if recruitment strategies actively targeted and increased the motivation for change before starting the intervention.

Yet, the between-group effect size *d* of 0.28 was higher than the minimal important difference defined as a standardized mean difference of 0.24, pinpointing the cutoff of clinical relevance in depression treatment [77]. Thus, Get.Back may be a promising treatment for this burdened population. To investigate the beneficial effects of Get.Back, a larger trial with sufficient power is needed on enhancing the overall treatment effect.

### Limitations

First, our findings should be interpreted as that of a pilot trial with limited power. Initially, the study was planned as an RCT with a total sample of 250 participants and was designed to specifically explore effects on return to work and cost-effectiveness of Get.Back. Our small sample size reduced the power to detect medium effect sizes. Second, the sample characteristics may have also limited the generalizability of our findings. The percentage of well-educated women was higher than in the general chronic pain population. Third, participants were recruited from a health insurance company. Therefore, results may not be generalizable to other settings in routine, clinical mental health care. Fourth, no clinical interviews took place at posttreatment or the 6-month follow-up. Therefore, changes in the diagnosis of MDD could not be analyzed. Future trials should therefore investigate the potential beneficial effects of the intervention with an extended follow-up period and with sufficient power. Fifth, the WAI version that was used in this study was not adapted for the use of internet interventions. Hence, exploring the agreement on goals might be difficult, as the goals are typically set by the intervention. An adapted version of WAI [78] for use in the internet interventions has been released and should be used in future studies.

### Implications for Clinical Practice and Recommendation for Future Research

Our findings have several implications for clinical practice. First, the results of this study suggest that a combined psychological treatment for patients on sick leave with comorbid recurrent depression and CBP might be beneficial. Available treatments generally only focus on one condition, rarely on both. Results of our study show that combining treatments for both conditions within one IMI appears to be feasible with high user satisfaction and acceptable adherence. However, although we found significant effects with respect to the primary outcome, the intervention was not found to be superior with regard to a range of secondary outcomes. It is unclear whether this finding is a result of the low power in this study or due to minimal efficacy for psychological interventions targeted at individuals with comorbid depression and CBP on sick leave. Hence, future studies are needed to compare different treatment modalities (eg, IMI vs f2f) with regard to both effectiveness and reach in the target group, given the challenging sample characteristics.

Second, using health insurance data to address individuals with CBP and a history of depression appears to be a promising strategy to reach individuals in need of treatment, as shown by the initial response rate of 5.5%. However, the rate of actual enrollment (76/6000, 1.26%) is lower than the response rates found in studies aimed at reducing mild to moderate depression and absenteeism in individuals at high risk for taking depression-related sick leave [73]. The requirement that individuals *opt in* to a study based on a postal invitation may have compounded difficulties of recruiting participants with depression.

Moreover, reacting to an invitation letter (eg, completing a baseline assessment and providing informed consent) may have been too demanding for individuals with severe depression. Individuals with depression might be interested in participating in internet-based interventions but not in a clinical trial. This may be particularly pertinent for individuals with CBP and comorbid depression. Consequently, future research should implement measures to reduce participant burden.

Another strategy to further enhance the potential of recruitment via a health insurance company could be the implementation of acceptance facilitation interventions (AFIs). The effectiveness of AFIs has been evaluated in recent research [79-81]. Such interventions may aim to increase the utilization of treatments by directly addressing potential barriers (eg, low outcome expectancy and fear of stigma). Future studies should therefore focus on improving initial response rates to health care insurance letters in addition to increasing conversion rates following expressed interest.

Third, the total cost (2683.20€; US $2973.79) [82], cost for initial response (8.05€; US $ 8.92) [82], and cost per included participant (35.30€; US $39.12) [82] were low. Cost per included participant was comparable with studies using Facebook ads (US $51.70; 46.64€) [82,83]. Compared with the high cost associated with non- or delayed treatments for multimorbid patients with chronic disease and depression, this cost is negligible. A meta-analysis concluded that it is difficult to assess the overall effectiveness of any particular recruitment strategy as some strategies that work well for a certain population may not be optimal for another population; they also discussed the necessity of additional research to better understand effective recruitment strategies [84]. For our studied population, the current recruitment strategy via health insurance letter invitations appeared feasible, but more research is needed to understand how response rates in untreated individuals with CBP and comorbid depression can be increased.

### Conclusions

To the best of our knowledge, this is one of the first RCTs investigating the effects of a psychological intervention in individuals with comorbid depression and CBP on sick leave. Despite our inability to examine the actual effects on return to work rates and cost-effectiveness of Get.Back, this trial shows that this particular group of individuals may benefit from IMIs, as shown by the positive user satisfaction ratings. However, besides larger follow-up confirmatory trials, future studies should implement strategies that could better reach the target sample, test possibilities to increase intervention effects, and identify subgroups of patients that may or may not benefit from such interventions and could otherwise be referred to other treatment modalities.

## Appendix

Multimedia Appendix 1 Overview of the Get.Back intervention, summary of content of each module, and screenshots of the intervention.

Multimedia Appendix 2 CONSORT-eHEALTH checklist (V1.6.1).

## Abbreviations

AFIs: acceptance facilitation interventions
AQoL-6D: Assessment of Quality of Life
CBP: chronic back pain
CBT: cognitive behavioral therapy
CES-D: Center for Epidemiological Studies Depression Scale
CSQ: Client Satisfaction Questionnaire
DSM: Diagnostic and Statistical Manual of Mental Disorders
f2f: face-to-face
FAU: Friedrich-Alexander-Universität Erlangen-Nürnberg
IMIs: internet- and mobile-based interventions
INEP: Inventory for the Assessment of Negative Effects of Psychotherapy
LOCF: last observation carried forward
MDD: major depressive disorder
MDQ: Mood Disorder Questionnaire
NNT: numbers needed to treat
QIDS-SR16: Quick Inventory of Depressive Symptomatology Self-Report
RCT: randomized controlled trial
SAE: serious adverse events
SCID: Structured Clinical Interview for the Diagnostic and Statistical Manual of Mental Disorders
TAU: treatment-as-usual
TiC-P: Trimbos and iMTA Questionnaire for costs associated with psychiatric illness
WAI: working alliance
WLC: waitlist control group
