# Supplementary material for: A Web- and Mobile-Based Intervention for Comorbid, Recurrent Depression in Patients With Chronic Back Pain on Sick Leave (Get.Back): Pilot Randomized Controlled Trial on Feasibility, User Satisfaction, and Effectiveness
Source: JMIR Ment Health. 2020 Apr 15;7(4):e16398. doi: 10.2196/16398 (PMC7191351; doi:10.2196/16398)
Supplement: Multimedia Appendix 1 [file mental_v7i4e16398_app1.pdf]

Multimedia-Appendix 1

Pilot RCT on feasibility, user satisfaction and effectiveness of Get.Back: a web- and mobile-based intervention for comorbid, recurrent depression in chronic back pain patients on sick leave

Authors:

Sandra Schlicker[1,2]; Harald Baumeister[3], Claudia Buntrock[1]; Lasse Sander[4]; Sarah Paganini[4]; Jiaxi Lin[4]; Matthias Berking[1]; Dirk Lehr[5]; David Daniel Ebert[1,6]

[1] Friedrich-Alexander University Erlangen-Nürnberg, Erlangen, Germany
[2] Philipps-University Marburg, Marburg, Germany
[3] Ulm University, Ulm, Germany
[4] Albert-Ludwigs-University Freiburg, Freiburg, Germany
[5] Leuphana University, Lüneburg, Germany
[6] Vrije University Amsterdam, Amsterdam, the Netherlands

Corresponding Author:
Sandra Schlicker
Friedrich-Alexander University Erlangen-Nürnberg
Department of Clinical Psychology and Psychotherapy
Naegelsbachstraße 25a
91052 Erlangen
Tel.: +49 (0)9131 85 67564
Fax: +49 (0)9131 85 67576
E-Mail: Sandra.Schlicker@fau.de

FRIEDRICH-ALEXANDER
UNIVERSITÄT
ERLANGEN-NÜRNBERG

PHILOSOPHISCHE FAKULTÄT
UND FACHBEREICH THEOLOGIE

Get.Back: IMI for comorbid Depression in chronic back pain patients on sick leave

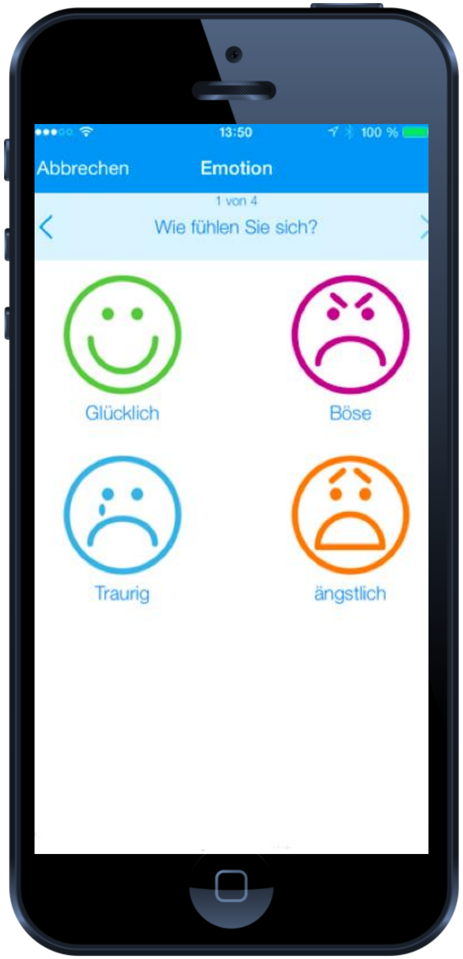

- weekly online modules
- 9 session and 1 booster session
- including home work assignements, interactive elements (emails, text messages, videos), reminders and excersises
- guidance by a trained psychologist (e-Coach) with weekly feedback on the sessions

- Psychoeducation
- Behavioral Activation
- Problem- Solving
- Ending rumination
- My way back to work
- Mood, Movement &Self-Esteem
- My plan for the future
- Healthy sleep Partnership & Intimacy — OPTIONAL
- Perfectionism Appreciation Social support Communication — MINI MODULE

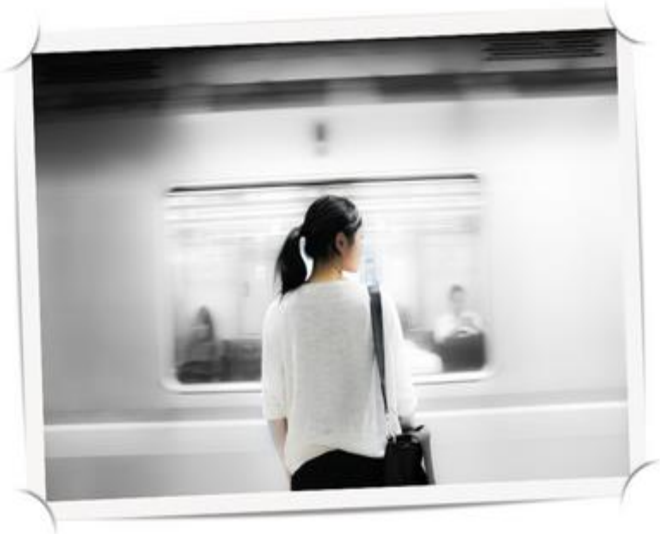

Module 1: Let‘s go!

You learn interesting facts about the **connection** between **back pain** and **depressive mood**. You learn what **acceptance** is with regard to your pain and why it might be important.

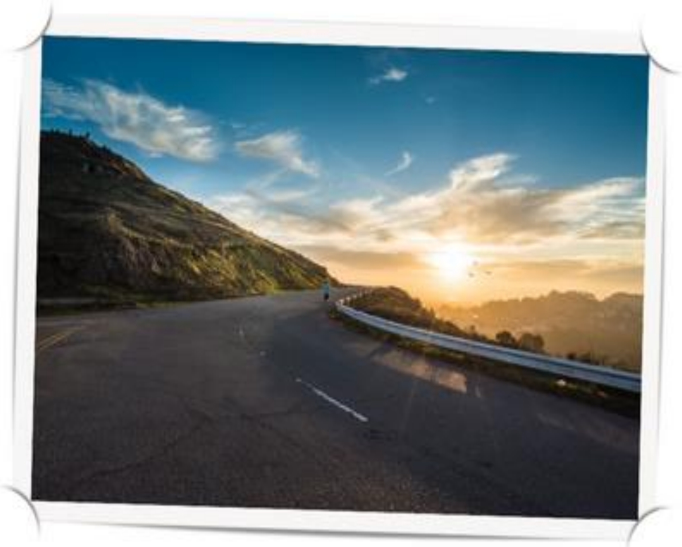

Module 2: Let‘s get active!

Get to know the **connection** between **activities** and **mood** and actively improve your mood.

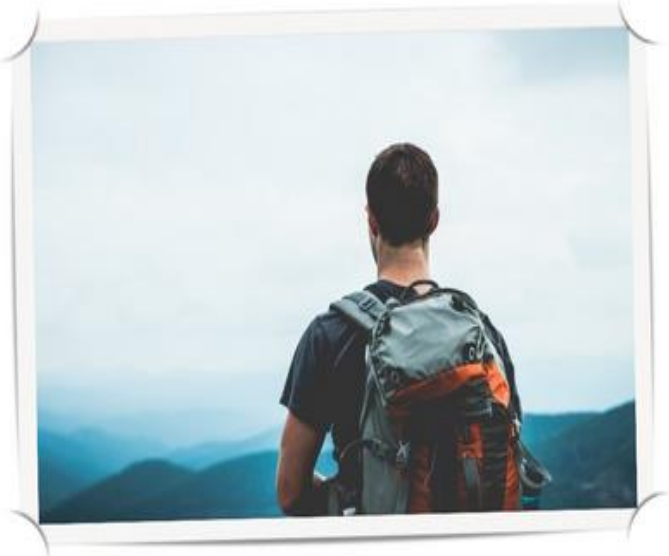

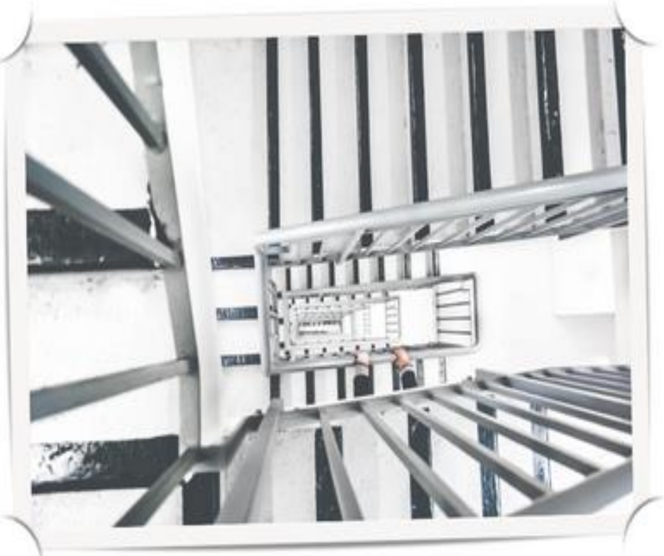

Module 3: Problem-Solving

What is a possible way from a **problem to** the **solution**? With the **6-step plan** you can try it out yourself in this lesson.

Module 4: Ending rumination

Learn how to deal with **rumination** and pain thoughts. You learn what **mindfulness** is and how to use it.

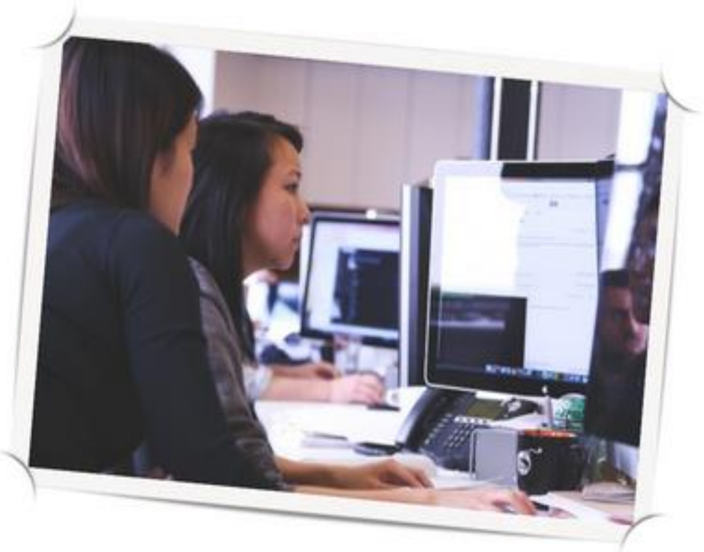

Module 5: My way back to work

This module is about coping with the **challenges** of **returning** to your **job**.

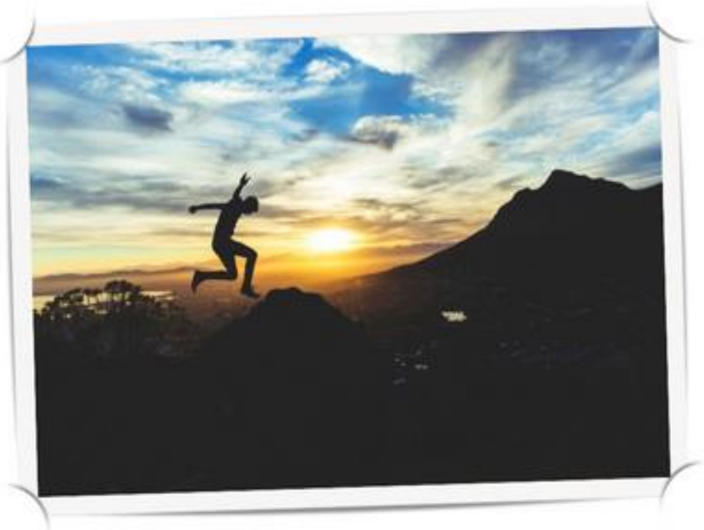

Module 6: Mood, movement & self-esteem

You learn how the connection between **mood** and **movement** can relate to your **self-esteem**. You learn about important components of your self-esteem and how to **improve** it.

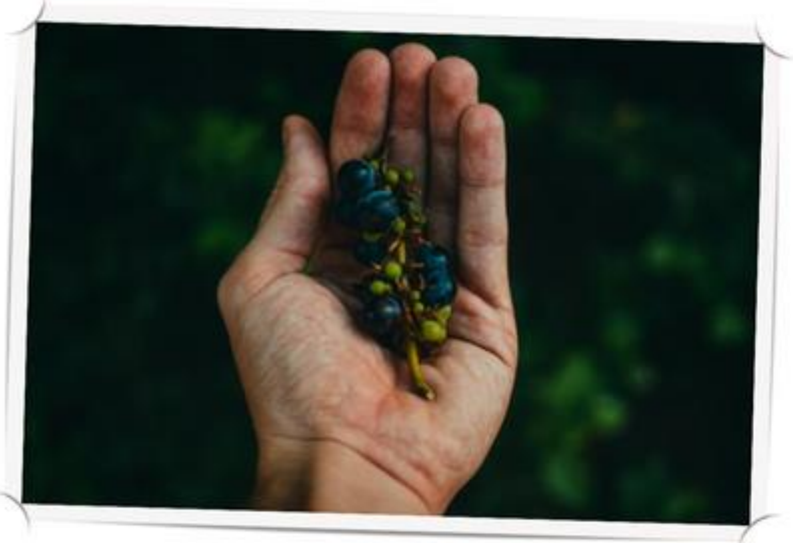

Module 7: My plan for the future

The training is **summarized** for you and you **plan your future** steps.

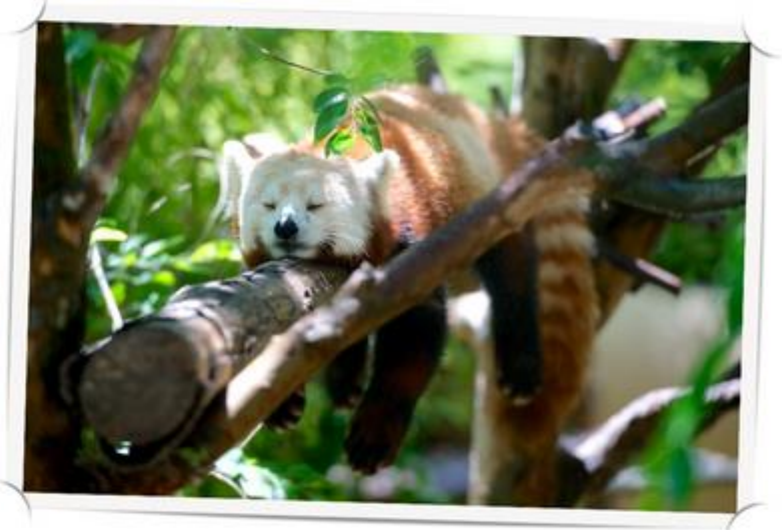

Optional: Healthy sleep

You learn what is important for **healthy sleep** and how to **deal** with **sleep problems**.

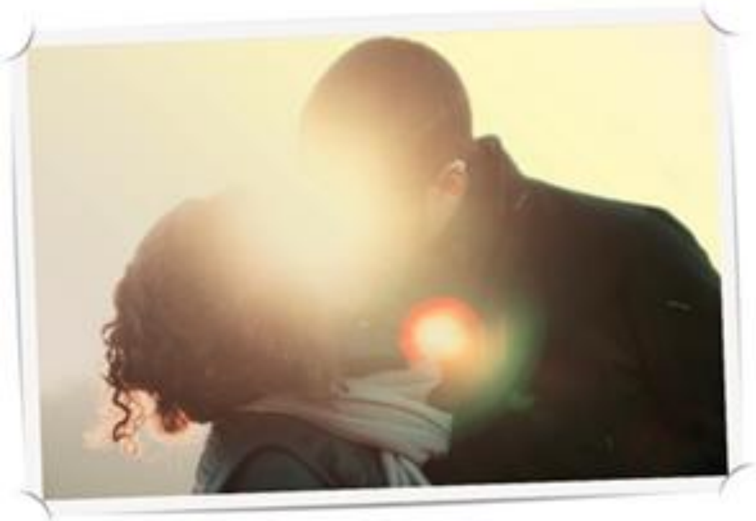

Optional: Partnership & Intimacy

You learn about **communication** and **intimacy** in a **partnership**.

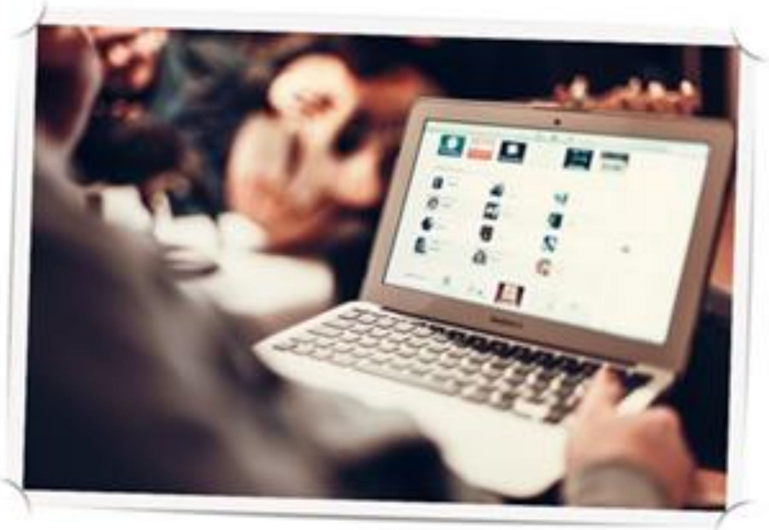

Booster Module

This module follows a four weeks after the end of the training and serves to **consolidate the contents**. Important content is repeated.

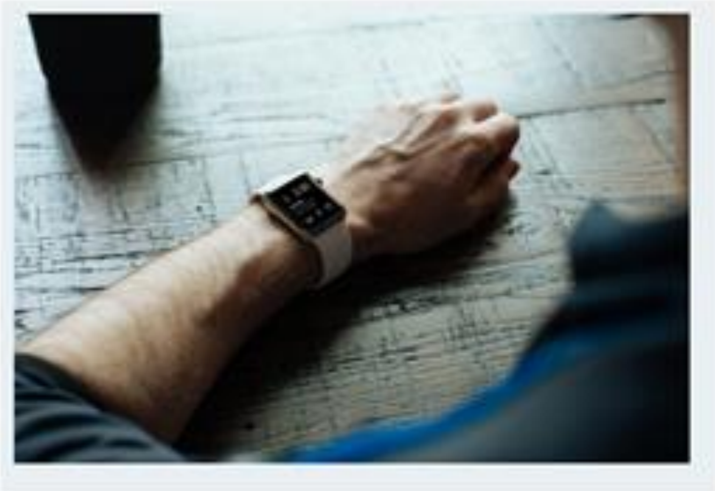

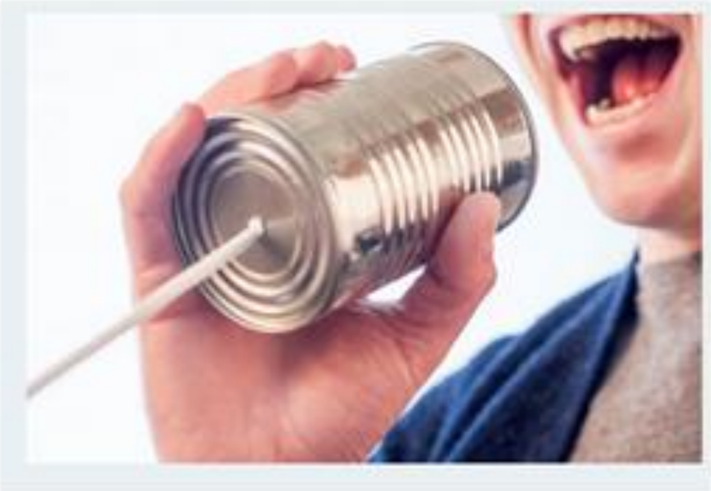

Mini-Module: Perfectionism

Learn about the **effects** of **perfectionist attitudes** and how you can use five rules to **change perfectionist principles**.

Mini-Module: Communication at work

You get to know about **aspects** of **communication** and receive information about **communication rules** in a **workplace** environment.

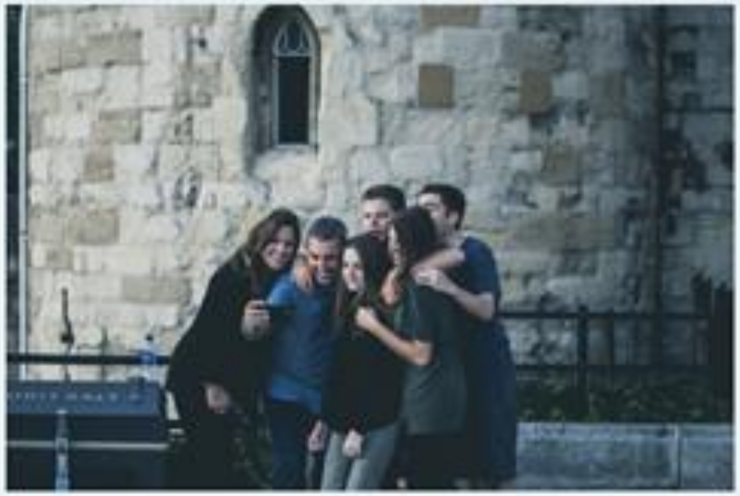

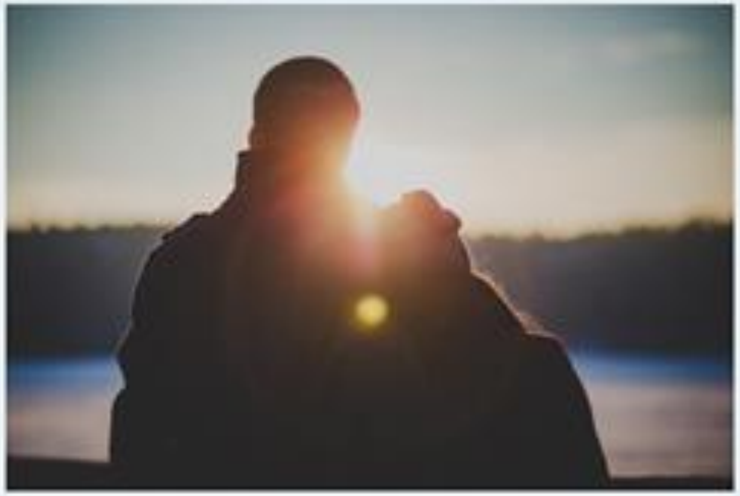

Mini-Module: Social Support

Learn more about how you can **receive** and **accept social support**. You also learn four steps to **self-confidence**.

Mini-Module: Appreciation

You receive information about what **"appreciating what is good"** is and experience in practical **exercises** how to **appreciate the good** in your life.

1. Wissenswertes über Arbeitsunfähigkeit, Stress und Depression

Text anhören

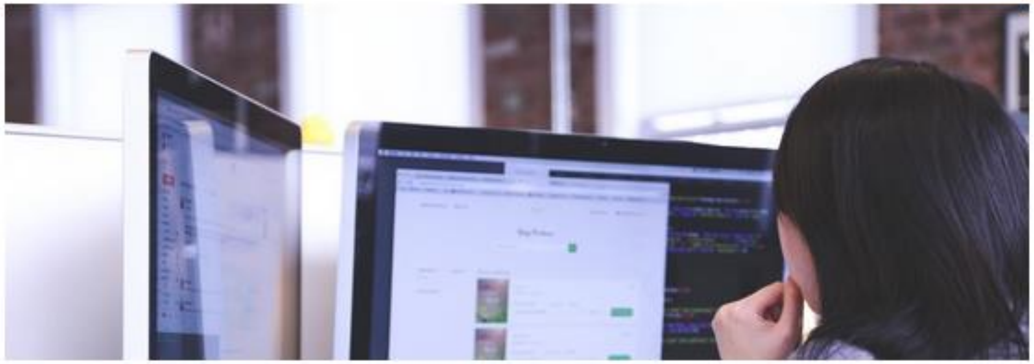

Arbeitsunfähigkeit

Wenn sich depressive Symptome entwickeln und wir gestresst sind, kann die Folge eine eingeschränkte Arbeitsfähigkeit sein. Eine beliebte Lösung ist eine Krankschreibung. Diese kann - kurzfristig - Abhilfe schaffen, in dem sie uns ermöglicht, uns zu erholen.

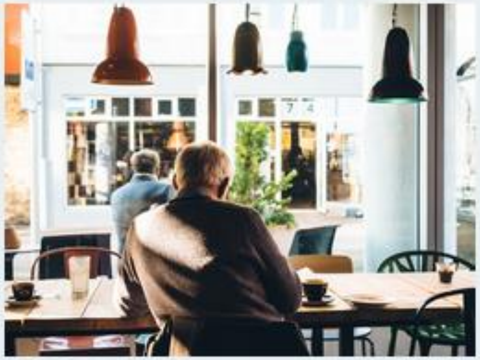

Den Teufelskreis durchbrechen

Langfristig kann sich jedoch eine Abwärtsspirale entwickeln:

Durch die plötzlich fehlende Struktur fühlen wir uns - nach anfänglicher Erholung - schnell unnütz. Neben der fehlenden Struktur stellt sich häufig auch ein Verlust von Sozialkontakten und Aufgaben ein. Damit einhergehend tritt häufig das Gefühl auf, nichts geschafft zu haben. Diese Faktoren begünstigen ein Stimmungstief. Um diesem zu entgehen, kann eine Rückkehr an den Arbeitsplatz wichtig sein.

Daher ist es wichtig, dass Sie sich aktiv für eine Rückkehr an den Arbeitsplatz entscheiden.

Start | 16 Aufgaben | Kontakt | Profil

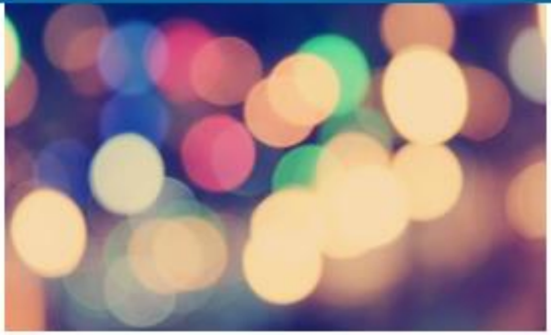

▾ Mehr lesen

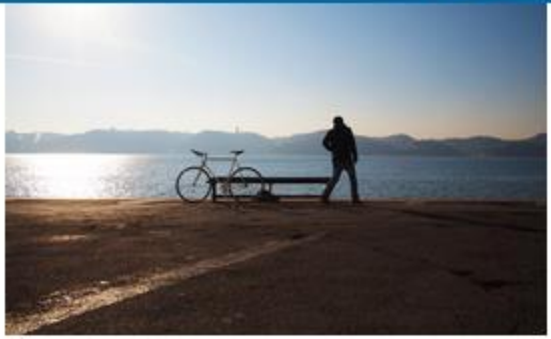

▾ Mehr lesen

Perfektionistische Leitsätze

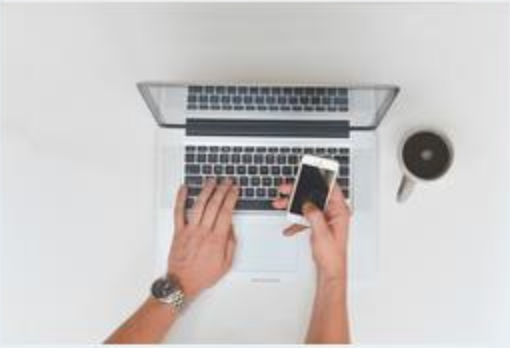

Ich muss, Ich kann nicht ...

Im Berufsalltag machen wir jeden Tag neue Erfahrungen.

Einstellungen und Verhaltensweisen, die früher erfolgreich waren, werden so zu Grundannahmen, von denen man sich kaum mehr distanzieren kann.

Auch perfektionistischem Verhalten liegen meist Leitsätze zugrunde, die uns zur zweiten Haut geworden sind und nur noch schwer aus dem Kopf zu bekommen sind.

Sie haben folgende perfektionistischen Grundannahmen bei sich festgestellt:

"Ich muss immer alles richtig machen."
"Ich muss kompetent sein."
"Ich muss perfekt sein."
